# An Expressway ETC Missing Data Restoration Model Considering Multi-Attribute Features

**DOI:** 10.3390/s23218745

**Published:** 2023-10-26

**Authors:** Fumin Zou, Zhaoyi Zhou, Qiqin Cai, Feng Guo, Xinyi Zhang

**Affiliations:** 1 Fujian Key Laboratory for Automotive Electronics and Electric Drive, Fujian University of Technology, Fuzhou 350118, China; fmzou@fjut.edu.cn (F.Z.); 18834603451@163.com (Z.Z.); n180310004@fzu.edu.cn (F.G.); 2231901001@smail.fjut.edu.cn (X.Z.); 2 School of Mechanical Engineering and Automation, Huaqiao University, Xiamen 361021, China; 3 College of Computer and Data Science, Fuzhou University, Fuzhou 350108, China

**Keywords:** ETC data, data restoration, missing transactions, expressway, data mining

## Abstract

Electronic toll collection (ETC) data mining has become one of the hotspots in the research of intelligent expressway extension applications. Ensuring the integrity of ETC data stands as a critical measure in upholding data quality. ETC data are typical structured data, and although deep learning holds great potential in the ETC data restoration field, its applications in structured data are still in the early stages. To address these issues, we propose an expressway ETC missing transaction data restoration model considering multi-attribute features (MAF). Initially, we employ an entity embedding neural network (EENN) to automatically learn the representation of categorical features in multi-dimensional space, subsequently obtaining embedding vectors from networks that have been adequately trained. Then, we use long short-term memory (LSTM) neural networks to extract the changing patterns of vehicle speeds across several continuous sections. Ultimately, we merge the processed features with other features as input, using a three-layer multilayer perceptron (MLP) to complete the ETC data restoration. To validate the effectiveness of the proposed method, we conducted extensive tests using real ETC datasets and compared it with methods commonly used for structured data restoration. The experimental results demonstrate that the proposed method significantly outperforms others in restoration accuracy on two different datasets. Specifically, our sample data size reached around 400,000 entries. Compared to the currently best method, our method improved the restoration accuracy by 19.06% on non-holiday ETC datasets. The MAE and RMSE values reached optimal levels of 12.394 and 23.815, respectively. The fitting degree of the model to the dataset also reached its peak ($R^{2}$ = 0.993). Meanwhile, the restoration stability of our method on holiday datasets increased by 5.82%. An ablation experiment showed that the EENN and LSTM modules contributed 7.60% and 9% to the restoration accuracy, as well as 4.68% and 7.29% to the restoration stability. This study indicates that the proposed method not only significantly improves the quality of ETC data but also meets the timeliness requirements of big data mining analysis.

## 1. Introduction

ETC systems are a well-established piece of infrastructure on expressways nowadays. As of the end of 2022, there were 27,000 ETC gantries nationwide, serving a user base of 260 million [1]. Being the largest internet-of-vehicles system globally, the widespread adoption of ETCs has garnered a vast amount of transaction data. Based on this data, scholars both domestically and internationally have undertaken a series of studies on the extension applications of intelligent expressways. The main contents include traffic parameter analysis [2,3], traffic condition estimation [4], traffic demand visualization [5], abnormal data detection [6], and traffic congestion identification [7]. It is evident that ETC data mining is a current research hotspot in the traffic field. However, since ETC gantries generally operate outdoors, they are susceptible to adverse environmental influences, potentially leading to equipment malfunctions. Meanwhile, as on-board units (OBU) pass through the gantries, phenomena such as wireless interference and obstructions caused by nearby large vehicles may occur. These issues can cause information exchange failures between devices, generating abnormal data. An assessment of the data quality of about 69,524,770 pieces of real transaction data collected from the Fujian Province ETC system on 1–5 May 2021 and 1–5 June 2021, revealed that the abnormal rate of ETC transaction data reached 7.71% [1]. These abnormal transaction data not only affect the operation and management of expressways but also increase the cost of data preprocessing, resulting in a significant deviation between the research results of relevant expansion application topics and the expected effects. Therefore, restoring the abnormal transaction data to ensure data quality is not only beneficial for accelerating the construction of intelligent expressways and achieving fine management of the transportation industry but also provides solid data support for the extended application of ETC big data mining, which is of great significance.

ETC transaction data are a kind of typical structured data. Presently, for the restoration of structured data, researchers often favor tree-based machine learning models. However, due to the large volume and complex feature composition of ETC transaction data, traditional machine learning models cannot precisely identify its data patterns, resulting in generally low restoration accuracy. On the other hand, with the deepening of research in big data mining, deep learning has demonstrated immense potential for applications in multiple fields. While the general framework of deep learning tends more to handle continuous data, the research on dealing with structured data is still in the initial stage, especially for categorical features, but this does not rule out the possibility of improvement and application. It is worth noting that most current research on data restoration concentrates on the restoration of chronological order, with very few focusing on time-quantitative restoration. Therefore, this paper summarizes the research work in the data restoration field in recent years and proposes an ETC missing transaction data restoration method based on the characteristics of ETC data. The main contributions are as follows:

(1) We propose a deep learning model based on multi-attribute feature analysis, which is capable of quantitatively restoring the transaction time of expressway ETC missing transaction data with high precision.
(2) Through analyzing data anomaly situations, we transformed the time restoration problem into an estimation problem of the vehicle’s section travel time, extracting key features from four perspectives.
(3) To better handle structured ETC data, we employ EENN to automatically learn the representation of classification features in multidimensional space, and utilize LSTM to extract the changing patterns of vehicle speeds over several consecutive sections, providing the model with more contextual information.
(4) Our method significantly enhances the restoration effects, improving the MAE and RMSE values by 19.06% and 10.66%, respectively, on non-holiday datasets, and demonstrating the best comprehensive performance on holiday datasets.

The paper is structured as follows: Section 2 overviews and summarizes related works. Section 3 elaborates on the methodology. Section 4 discusses experimental results and analysis. The final section concludes the work and looks forward to future prospects.

## 2. Related Work

As a hot topic and a key research area in data mining, many experts and scholars have made efforts in the field of data restoration. Currently, the main data restoration methods include statistical-based restoration methods, relational-based restoration methods, and learning-based restoration methods.

Initial studies on missing data restoration focused on statistical-based restoration methods, which mainly used the average value of historical data for data interpolation, referred to as historical mean interpolation. This method used the entire dataset for missing interpolation, in which some unrelated data might affect the accuracy or complexity of the interpolation. Based on different data processing needs, there are mainly two types: local average [8] and global average [9]. The historical mean imputation method generally performs poorly in most case, hence it has not received much attention and there are not many related studies. With the deepening of research, scholars have found that it is possible to restore missing data by modeling the data to be restored based on the correlation between data attributes, and estimating the missing data according to the model. Rahman et al. [10] made a preliminary attempt, considering some attributes that are highly correlated with the attributes with missing conditions, ignoring all other attributes with low correlation, and combining with similarity analysis to fill in missing values. This method indeed improved the accuracy of the restoration, but it increased the complexity of the model. Principal component analysis (PCA) is a common method in statistical analysis. It can reduce the complexity of the model without losing too much data information by reducing the dimensionality of the data [11]. There are some relevant studies based on this method and its improved methods. Representative methods mainly include functional principal component analysis (FPCA) [12], probabilistic principal component analysis (PPCA) [13], and kernel probabilistic principal component analysis (KPPCA) [14], among others.

It is evident that statistical-based restoration methods can to a certain extent reflect some correlations existing internally within the data. However, this approach possesses high complexity. To address this, some scholars have introduced relational data restoration methods, which either delineate data consistency utilizing functional dependencies and conditional constraints or convert data into matrix and tensor expressions, all capitalizing on the correlation between data attributes [15,16,17,18,19,20,21]. Early research generally utilized functional dependencies to directly describe data consistency [22,23]. There are also scholars who have attempted to add extra constraints on the basis of functional dependencies, achieving good results [24,25]. The method of functional dependence combined with constraints is often used to solve strategy optimization problems for urban travel demand [26]. Due to the strict equality relationships required by functional dependencies and rule constraints, it is difficult to discover absolute consistency in real time series datasets. To this end, Gao et al. [27] proposed a time series restoration method based on multi-interval velocity constraints. Related studies indicate that, for the restoration issues pertaining to time series data, having accurate timestamps ensures satisfactory restoration effects. It is evident that for time series data, accurate timestamps are extremely vital. Regrettably, the research on timestamp restoration is still immature, resulting in scant related literature. Duan et al. [28] developed a method based on graph models for the discovery of time-sensitive rules and the restoration of data sequences.

In recent years, machine learning has garnered considerable attention. There has also been research leveraging machine learning models for data restoration through prediction, known as learning-based restoration methods. In the data restoration domain, machine learning algorithms demonstrating outstanding performance include the clustering algorithm (CA) [29,30,31,32,33], XGBoost algorithm [34], random forests (RF) [35], and neural networks (NN) [36,37,38,39,40], among others. The CA can separate markedly missing data from the dataset based on similarity, hence some studies have undertaken missing data restoration relying on the results of clustering [29,30]. However, the basic CA rigidly classifies data into certain categories, which does not align with real-life scenarios. Therefore, some scholars have introduced the fuzzy c-means (FCM) algorithm. This method assigns weights to data categories to indicate the degree to which data belong to a certain category, offering more flexible clustering outcomes. Related research shows that compared to the basic CA, the FCM algorithm significantly improves restoration accuracy [31]. These methods all work based on the results of clustering to carry out data restoration tasks, hence to some extent, the choice of clustering-related parameters affects the effectiveness of data restoration. To address this, some researchers have employed strategies such as particle swarm optimization (PSO) and the genetic algorithm (GA) to optimize related parameters, achieving an enhancement in restoration accuracy. In 2016, Duan et al. [36] pioneered the use of deep learning in addressing missing traffic data issues. The study indicates that deep learning holds considerable potential in traffic data supplementation, urging the continued development and exploration of innovative deep learning structural models for interpolation and their practical application. Building on this, several scholars have successively conducted research on deep learning-based data restoration algorithms. Fan et al. [37] compared three models for predicting data with varying missing rates, finding that the general regression neural network (GRNN) exhibited strong robustness for data with higher missing rates. Zhang et al. [38] proposed a traffic flow data restoration model based on self-attention mechanisms and graph autoencoders, which learns the urban road network topology through self-attention mechanisms and captures temporal regularities and spatiotemporal correlations in the data using LSTM networks combined with attention mechanisms. Kazemi et al. [39] introduced an iterative generative adversarial network (IGAN) for missing data interpolation. Hou et al. [40] proposed a graph convolution restoration method that integrates traffic flow spatiotemporal features. Other scholars have also made efforts with different methods, achieving good restoration results [41,42,43]. We have summarized these related works, and elaborated on the advantages and disadvantages of these methods, as detailed in Table 1.

After conducting a comprehensive analysis of existing data restoration methods, we found that many methods face challenges in capturing the complex non-linear relationships within the data. Furthermore, these methods often require strict parameter settings and regular selection, which might lead to unstable restoration accuracy. Due to the extensive deployment of ETC systems on expressway networks, ETC data encompass a myriad of complex vehicle driving patterns. Concurrently, being a real dataset, ETC data are produced by various interactive rules and constraints. These issues undoubtedly increase the difficulty of data restoration. Although researchers have attempted to restore ETC data [34,35], these methods still have limitations. Specifically, the former focuses on restoring the derivative statistical data of ETC rather than the original data, while the latter is limited to restoring data on specific expressway sections, lacking universality. Deep learning methods can not only effectively handle large volumes of ETC data with complex patterns, but also reduce the time complexity without the need for repetitive model training. Most research tends to use deep learning to process unstructured data such as GPS, video images, and weather. But since ETC data are structured data containing various attribute types, its restoration requires an integrated method. Therefore, we employed a combination of various deep learning models, selecting appropriate models for each data attribute type, to achieve efficient data restoration.

## 3. Methodology

**Figure 1:**
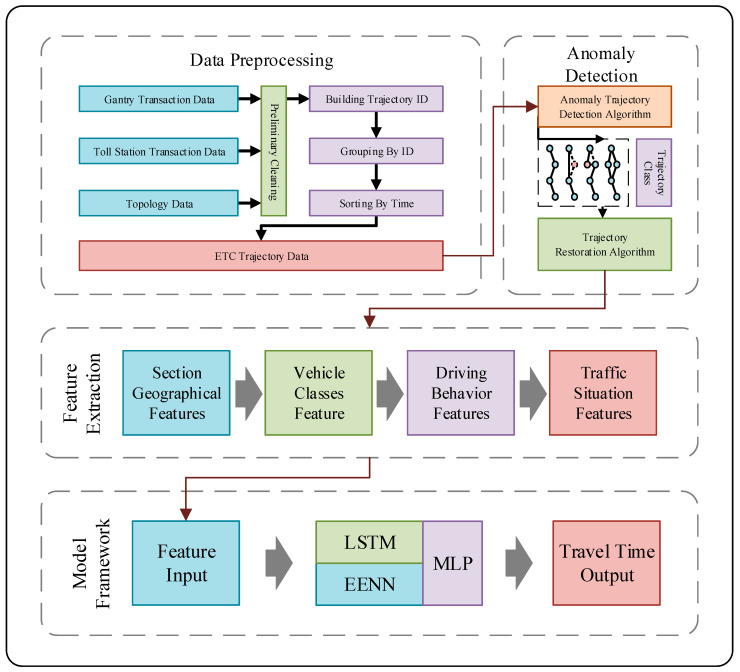
Overall architecture of the method.

To address the issue of ETC missing transaction data time restoration, we propose a deep learning model based on multi-attribute feature analysis. First, we preprocess the ETC data. Subsequently, we classify the trajectory data using an anomaly trajectory detection algorithm. Then, we extract the relevant features. Finally, we use these features as the inputs for the model. The overall architecture of this method is shown in Figure 1.

### 3.1. Definitions

To facilitate the description and understanding of this paper, the following related definitions are provided in this section:

ETC trajectory data: when a vehicle equipped with an OBU device is driving in the expressway network, it engages in dedicated communication with the toll station or ETC gantry it passes, generating transaction information records. According to the positions of the gantries and toll stations provided by the road network topology data, the driving trajectory of the vehicle on the expressway can be restored. These trajectories take toll stations as OD, encompassing a series of gantry nodes arranged in chronological order, referred to as ETC trajectory data.

Section: According to the layout of ETC road equipment in the road network, both toll stations and gantries can be regarded as nodes N, and a section consists of two adjacent nodes and the road range between them, hence we have the node expression:

(1) $$ExSec=\langle N_{1},N_{2}\rangle$$

in which $N_{1}$ denotes the gantry before the section, and $N_{2}$ represents the gantry after the section. If a service/parking area is present in the section, we call it a service area/parking area section.

Road: A road is referred to as a range composed of two or more continuous sections, and we have:

(2) $$\begin{array}{c} ExRos=\langle {ExSec}_{1},{ExSec}_{2},\ldots ,{ExSec}_{n}\rangle \\ =\langle N_{1},N_{2},\ldots ,N_{n+1}\rangle ,n\geq 2 \end{array}$$

in which the element ${ExSec}_{n}$ of the road is called the nth section, and n must be at least 2. The road definition can also use node expressions, with its components $N_{n}$ called the nth node. It should be noted that, unlike toll station nodes that only serve as the starting and ending points of a section, two adjacent sections share a gantry at the connecting point of the section.

Section/Road travel time: The time spent by a vehicle passing through a certain section/road, called the travel time of the vehicle in this section/road ∆t, is given by:

(3) $$∆t=T_{2}-T_{1}$$

in which $T_{1}$ is the transaction time at the front gantry of the section or the starting node of the road, and $T_{2}$ is the transaction time at the rear gantry of the section or the end node of the road. If m vehicles pass through this section/road during the ith time period, the travel time of these vehicles ∆T can be represented as:

(4) $$∆T=\left\{{∆t}_{i}^{1},{∆t}_{i}^{2},\ldots ,{∆t}_{i}^{m}\right\}$$

We call the average travel time of these m vehicles passing through this section/road during the ith time period the section/road travel time $\bar{\tau }$, which is:

(5) $$\bar{\tau }=\sum_{a=1}^{m}{∆t}_{i}^{a}/m$$

Section/Road speed: According to a vehicle’s section/road travel time ∆t and its section/road distance ∆d, the travel speed of the vehicle in this section/road v can be obtained using the speed formula:

(6) v=∆d/∆t

where the speed unit is km/h. Similarly, if m vehicles pass through this section/road during the ith time period, the travel speeds of these vehicles V can be represented as:

(7) $$V=\left\{v_{i}^{1},v_{i}^{2},\ldots ,v_{i}^{m}\right\}$$

We call the average speed of these m vehicles passing through this section/road during the ith time period the section/road speed $\bar{\nu }$, which is:

(8) $$\bar{\nu }=\sum_{a=1}^{m}v_{i}^{a}/m$$

### 3.2. Problem Descriptions

**Figure 2:**
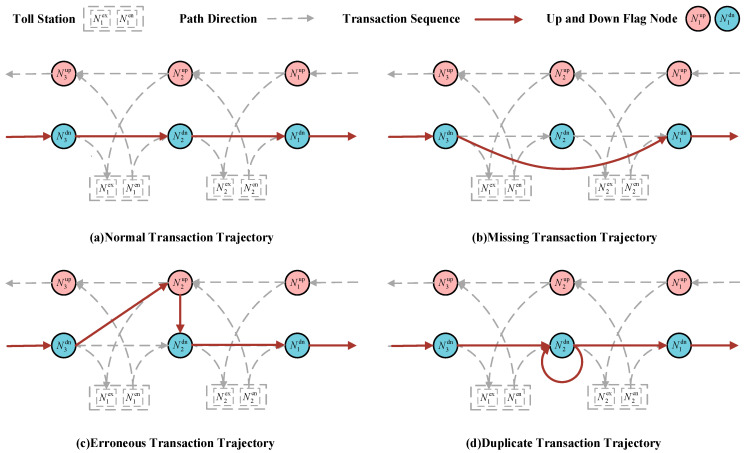
The gantry transaction order of different kinds of abnormal trajectory data.

Abnormal transaction events mainly include three types: missing transactions, erroneous transactions, and duplicate transactions [1]. Figure 2 displays the gantry transaction order of different kinds of abnormal trajectory data. The essence of erroneous and duplicate transactions is data redundancy, which can be directly deleted. The key issue to be addressed in this paper is the missing transaction data. According to statistics, the probability of having more than two consecutive gantries experiencing missing transactions is less than 5% [1]. Therefore, this paper mainly targets the majority of cases for restoration, hereby stipulating that in the missing transaction trajectory data discussed in the subsequent parts of the article, only one gantry is missing consecutively. This mean that there are normal transaction records for the two gantries adjacent to the missing gantry. According to the definition, in the missing transaction trajectory data, there exists a missing transaction road $ExRos=\langle {ExSec}_{1},{ExSec}_{2}\rangle =\langle N_{1},N_{2},N_{3}\rangle$, where the $N_{2}$ is the missing transaction gantry. From the definition of travel time, based on the transaction time $T_{1}$ of the vehicle at $N_{1}$ and the travel time of the ${ExSec}_{1}$ section ∆t, the transaction time $T_{2}$ at $N_{2}$ can be calculated as follows:

(9) $$T_{2}=T_{1}+∆t$$

Hence, restoring the transaction time issue of the missing transaction gantry translates to estimating the travel time in the section where the missing transaction gantry serves as the section’s end gantry. We can extract numerous driving characteristics of vehicles in sections from the ETC transaction data. Essentially, our restoration method is to mine the potential features of the data and reveal the intrinsic connections between data characteristics.

### 3.3. Data Preprocessing

Data preprocessing mainly consists of three parts: original data screening, trajectory data construction, and normalization.

The original ETC transaction data used in this paper mainly include ETC gantry transaction data, ETC toll station transaction data, and ETC topology data. Gantry transaction data mainly record the transaction information of the vehicle with the gantry and the transaction information of the toll station at the entrance of the road network. Toll station transaction data primarily record the transaction information of vehicles entering and exiting the toll stations in the road network. The topology data mainly record relevant information about the section. Firstly, we extract the necessary fields from the ETC transaction data as shown in Table 2.The vehicle identification in the ETC transaction data is mainly based on the registration information at the time of ETC handling or camera license plate recognition, so there may be phenomena of unsuccessful identification, leading to garbled fields such as obusn, vehclass, enstation, and exstation, and it is necessary to filter out these erroneous data based on the regular expression of field encoding, completing the original data cleaning.

Since some vehicles may enter and exit the expressway network multiple times in a day, generating multiple travel trajectories, we combine obusn, enstation, and enstation to form the vehicle trajectory identification field passid. Then, we group the ETC gantry transaction data according to passid and sort them by transaction time to obtain the vehicle gantry trajectories. As the expressway is a closed network, vehicles must use toll stations as network ODs while traveling on the expressway, so we need to use toll station transaction information to match the entrance and exit toll stations for gantry trajectories, forming complete closed ETC trajectory data. For some vehicle trajectories where the starting and ending nodes are not toll stations, they need to be screened out. Finally, based on the topological data, we need to match the relevant information for each section in the trajectory.

Before inputting the data into the model, it is necessary to separate the discrete and continuous data according to data attributes and normalize the continuous data as follows:

(10) $$x^{\prime }=\frac{x-x_{min}}{x_{max}-x_{min}}$$

where $x_{max}$ and $x_{min}$ represent the maximum and minimum values in the column where x is located, and $x^{\prime }$ is the normalized data of x. The data preprocessing algorithm is as shown in Algorithm 1.
**Algorithm 1** ETC Trajectory Data Preprocessing Algorithm**Input:** ETCGantryData, ETCTollStationData, ETCTopologyData**Output:** TrajectoryDataList1: Initialize TrajectoryDataList = []
2: **for** each vehicle **in** ETCTollStationData:
3:   vehicleID = vehicle.passid
4:   vehicleClass = vehicle.vehclass
5:   entryStation = vehicle.enstation
6:   entryTime = vehicle.entime
7:   exitStation = vehicle.exstation
8:   exitTime = vehicle.extime
9:   # Data Cleaning
10: **if** isCorrupted(vehicleID) **or** vehicleClass == 0 **or** isCorrupted(entryStation) **or** isCorrupted(exitStation):
11:  **Continue**
12: # Match gantry transactions
13: matchedGantryTransactions = matchGantryTransactions(ETCGantryData, vehicleID, vehicleClass, entryStation, entryTime)
14:  # Sort transactions by time
15:   sortedTransactions = sortByTransactionTime(matchedGantryTransactions)
16:  # Generate trajectory data
17:   trajectory = [entryStation] + sortedTransactions + [exitStation]
18:   timeSequence = [entryTime] + extractTimes(sortedTransactions) + [exitTime]
19:   # Data Cleaning
20:   **if** notIsTollStation(trajectory[0]) **or** notIsTollStation(trajectory[−1]):
21:    **Continue**
22:   Initialize speedList = []
23:   **for** i **in** range (0, len(trajectory) − 1):
24:    startPoint = trajectory[i]
25:    endPoint = trajectory[i+1]
26:    distance = getDistance(ETCTopologyData, startPoint, endPoint)
27:    timeDifference = timeSequence[i+1] − timeSequence[i]
28:    speed = calculateSpeed(distance, timeDifference)
29:    # Data Cleaning
30:    **if** speed > 180:
31:     **Continue**
32:    Append speed to speedList
33:   Append {“trajectory”: trajectory, “timeSequence”: timeSequence, “speed”: speedList} to TrajectoryDataList
34: End For each vehicle
35: **Return** TrajectoryDataList

### 3.4. Data Analysis and Feature Extraction

(1) Section Geographical Features

**Figure 3:**
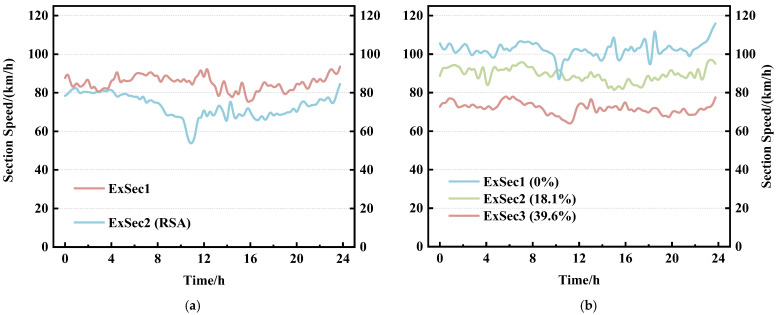
(**a**) Section speed variation curve for common sections and service area sections, where ${ExSec}_{1}$ represents common section and ${ExSec}_{2}$ represents service area section. (**b**) Section speed variation curve for three different sections; the tunnel lengths account for 0%, 18.1%, and 39.6% of the total section length, respectively.

The ETC gantry system divides the Fujian province expressway network into 2950 sections. Based on the statistical analysis of the expressway ETC topology dataset, in the sections solely constituted by gantry nodes, there exists a service area section for every 4 sections on average. As can be inferred from Figure 3a, the section speed of the service area sections is significantly lower than that of the common sections during certain periods, which is due to the possibility of vehicles entering the service areas for a stop. Therefore, the existence of a service area in a section can affect a vehicle’s section travel time Δt, further affecting the section speed $\bar{v}$ at certain times.

Related research indicates that vehicle speeds within tunnels are generally lower than the speeds on the roads before and after the tunnels [44]. As the number and length of tunnels in a section increase, the proportion of a vehicle’s tunnel travel state in the entire section’s driving state increases, potentially leading to a decrease in the vehicle’s section travel time Δt, further affecting the overall section speed $\bar{v}$, as can be seen from Figure 3b. Based on the above analysis, we construct the section geographical feature vector γ, which is defined as:

(11) $$\gamma ={\left(\gamma_{1}{,\gamma }_{2},\gamma_{3},\gamma_{4}\right)}^{T}$$

In which, $\gamma_{1}$ represents the section distance (unit: m). $\gamma_{2}$ represents whether the section contains a service area, with values of 0 (no service area) and 1 (service area present). $\gamma_{3}$ represents the number of tunnels in the section. $\gamma_{4}$ represents the total length of all tunnels in the section (unit: m).

(2) Vehicle Classes Feature

According to the recording method of the ETC system for vehicle classes, there are a total of 16 classes of vehicles. Vehicles are divided into three major categories based on their functions: passenger vehicles, freight vehicles, and special operation vehicles. These major categories are further subdivided into various subcategories based on passenger capacity, load-bearing, and operational types. Different classes of vehicles have different proportions in the expressway traffic flow. According to statistics, class 1 passenger vehicles, which means passenger vehicles with a seating capacity of nine or less, account for about 70% of the expressway traffic flow. Class 1 freight vehicles, which means those with two axles, less than 6 m in length, and a maximum allowable total weight of 4.5 tons, account for approximately 12% of the expressway traffic flow, while other classes of vehicles make up the remaining 18%. Due to the different speed limits imposed on different classes of vehicles on the expressway, coupled with differences in driving behaviors of different vehicles, their section travel time Δt also varies. Class 1 passenger vehicles and class 1 freight vehicles constitute a larger proportion in the traffic flow composition and can be analyzed separately. The proportions of class 2 and above passenger and freight vehicles, and all special operation vehicles, are too small. Excessive categorization would result in too small sample sizes for these classes of vehicles, making it difficult to fit the distribution.

**Figure 4:**
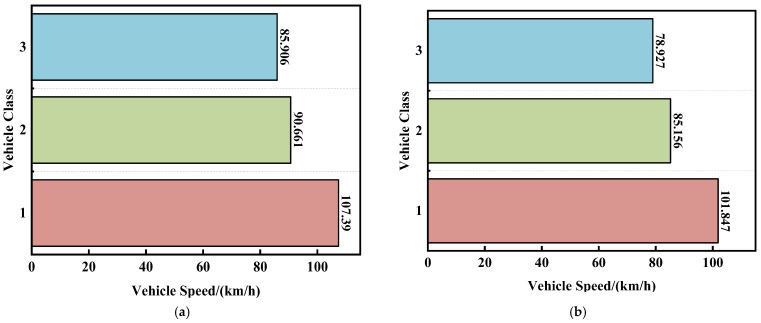
After the class division, the average travel speeds of the three kinds of vehicles over sections with lengths of 18,624 m (**b**) and 2633 m (**a**).

As shown in Figure 4, after the merging of vehicle classes, the three classes of vehicles show significant differences in their travel speeds over long and short distances of sections. Therefore, we construct vehicle classes feature vector ε, with the value ranges being 1 (class 1 passenger vehicle), 2 (class 1 freight vehicle), and 3 (other classes of vehicles).

(3) Driving Behavior Features

**Figure 5:**
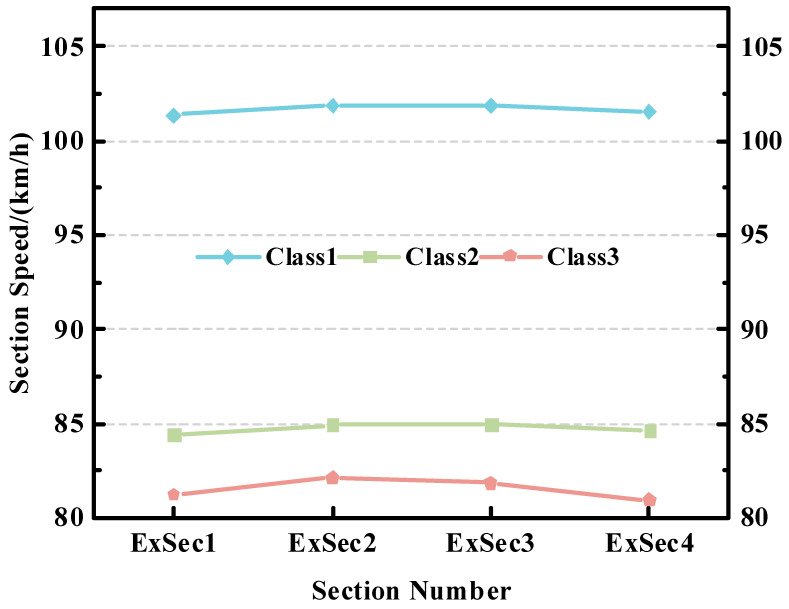
Fluctuation curves of section speeds for three different classes of vehicles traveling in the same consecutive four sections.

According to the driving habits on expressways, most vehicles maintain a small fluctuation range in speed over several consecutive sections. We can see from Figure 5 that only a few special operation vehicles show larger changes in speed. Therefore, there might be a correlation in the speed of vehicles over several contiguous sections.

Based on the analysis above, we construct the vehicle driving behavior feature η, which is defined as follows:

(12) $$\gamma ={\left(\eta_{1},\eta_{2},\eta_{3}\right)}^{T}$$

where $\eta_{1}$ represents the travel time of the vehicle in the missing transaction section, measured in seconds. $\eta_{2}$ and $\eta_{3}$ respectively denote the speeds of the vehicle in the section before and after the missing transaction road, measured in km/h.

(4) Traffic Situation Features

**Figure 6:**
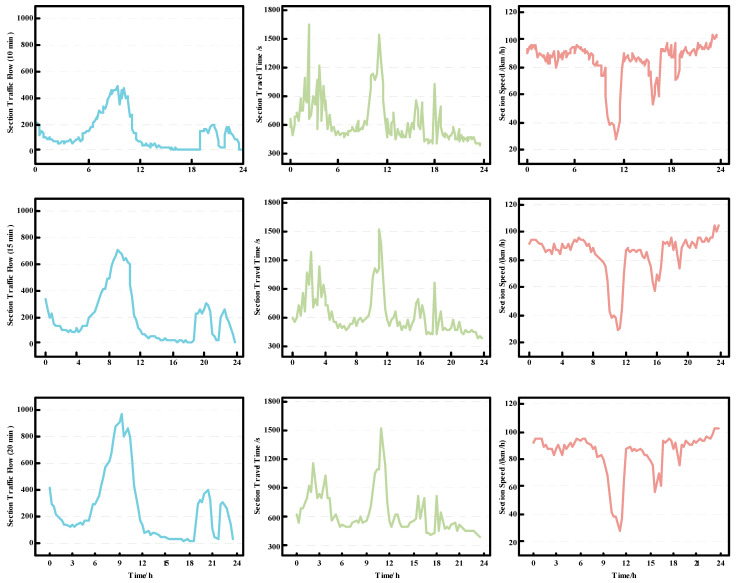
From top to bottom, left to right, the changes in section traffic flow, section travel time, and section speed curves over 24 h in a day are shown, with time slices of 10 min, 15 min, and 20 min.

The traffic situation of a section is mainly composed of the section flow, section travel time, and section speed, which affect each other. To accurately reflect the changes in the traffic situation of a section, we divided the whole day (0:00 to 24:00) into time slices of 10, 15, and 20 min, and performed corresponding statistics. As shown in Figure 6, compared to other time intervals, the 15-min time slicing method does not lose too many details of traffic situation changes. Moreover, this method does not overly refine the time segments, avoiding the situation where some low-flow sections have no vehicles passing through in some time slices, resulting in feature loss and complicating data processing and model effects.

We can observe from Figure 6 that the section flow fluctuation curve presents an ‘M’-shaped dual-peak state throughout the day, indicating higher vehicle volumes during morning and afternoon periods. Meanwhile, as the section flow increases to a certain threshold, the section speed shows a decreasing trend, leading to an increase in section travel time. This might be because the number of vehicles driving in this section during the same period exceeded the traffic carrying capacity of the section, resulting in traffic congestion. Because the traffic condition of the section also affects the travel time of the vehicle, we construct the traffic situation feature θ. It should be noted that, due to different travel times and speeds for different classes of vehicles, we selected vehicles of the same class when calculating the section travel time and section speed.

(13) $$\theta ={\left(\theta_{1},\theta_{2},\theta_{3},\theta_{4}\right)}^{T}$$

where $\theta_{1}$ denotes the time zone to which the vehicle belongs, dividing the 24 h into 96 time zones with 15-min slices, with a value range of [1,96]. $\theta_{2}$ indicates the section flow, which means the number of vehicles passing through that section in a certain time zone. $\theta_{3}$ and $\theta_{4}$ represent the section travel time and section speed, respectively, calculated for vehicles of the same class as the vehicle involved in the missing transaction data, measured in s and km/h.

### 3.5. Model Framework

The ETC transaction time restoration represents a typical structured data regression problem. Firstly, we transform it into an approximate function $\hat{y}$, as follows:

(14) $$\hat{y}=f\left(x_{1},x_{2},\ldots ,x_{n}\right)$$

**Figure 7:**
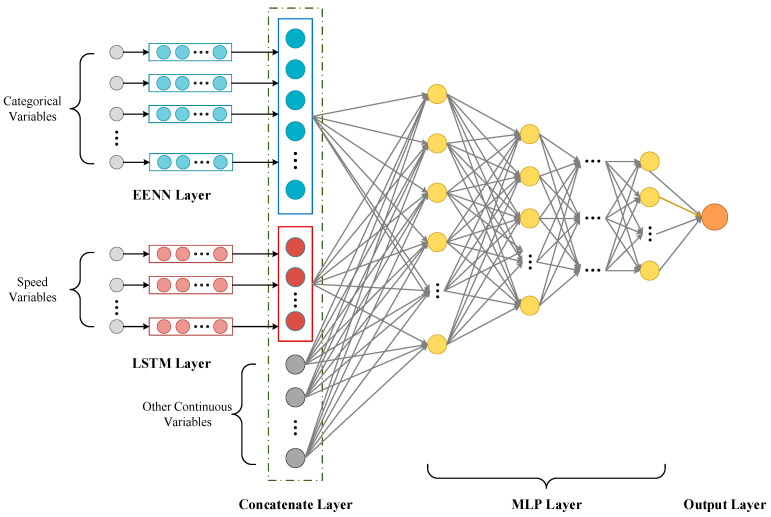
Model framework.

Given a set of input values $\left(x_{1},x_{2},\ldots ,x_{n}\right)$, which means a set of feature vectors, the target output value $\hat{y}$ is generated. Generally speaking, NN can approximate almost any function. However, because its general form assumes a certain degree of continuity, it is very unsuitable for approximating discontinuous functions [45]. The features reflecting the driving patterns of vehicles on expressways have a complex composition of attribute types. Feeding them directly into a deep learning model without appropriate processing might lead to suboptimal estimate outcomes. Specifically, categorical features often contain multiple categories. Without proper handling, or by solely using traditional one-hot encoding, this can result in high-dimensional and sparse feature vectors. That makes model training challenging, and may even lead to the curse of dimensionality. Moreover, continuous time-related data, like the driving speed across consecutive sections, often possess time series characteristics, and conventional deep learning models might not effectively capture their temporal dependencies. In response to these issues, we propose a combined deep learning model. As illustrated in Figure 7, the model framework mainly consists of three parts: EENN for handling discrete variables, LSTM for extracting speed variation features over several continuous sections, and MLP for receiving the processed variables, integrating them with other continuous variables, and outputting the estimated passage time for the vehicle’s missing transaction sections, further accomplishing time restoration.

(1) Categorical Features Processing Using EENN

Structured data with categorical features might lack continuity altogether, or even if present, it may not be so apparent. Therefore, traditional deep learning models do not particularly excel at handling such discrete variables. Moreover, categorical features often consist of a multitude of categories, and the conventional one-hot encoding method can lead to dimensionality explosion. Compared to conventional encoding methods, entity embedding can capture complex relationships within categorical variables at a much lower dimensionality. This is not just because it offers a dimensionality reduction strategy, but also because it provides “positions” for different categories within the embedding space, and these “positions” reflect their relative relationships. Additionally, the embedding vectors, being part of the neural network, can undergo end-to-end training, making them highly relevant to downstream tasks. By utilizing EENN, we automatically learn representations of categorical features in a multi-dimensional space. This approach not only reduces memory usage and accelerates the neural network, but more importantly, it reveals intrinsic properties of categorical variables by mapping similar values close to each other in the embedding space. This makes values with similar effects in Equation (14) close to each other. The working principle of the EENN is as follows.

Entity embedding initially maps each state of discrete variables to a vector:

(15) $$e_{i}\;:\;x_{i}⟼X_{i}$$

This mapping is equivalent to adding a layer of linear neurons on the one-hot encoded input. To illustrate this, we represent the one-hot encoding of $x_{i}$ as:

(16) $$u_{i}\;:\;x_{i}⟼\delta_{x_{i}\alpha }$$

where $\delta_{x_{i}\alpha }$ is the Kronecker delta, and α has the same possible values as $x_{i}$. If mi is the number of values for the categorical variable $x_{i}$, then $\delta_{x_{i}\alpha }$ is a vector with a length of $m_{i}$, where the element is non-zero only when ${\alpha =x}_{i}$.

Given the input $x_{i}$, the output of the additional linear neuron layer is defined as:

(17) $$x_{i}\equiv \sum_{\alpha }\omega_{\alpha \beta }\delta_{x_{i}\alpha }=\omega_{x_{i}\beta }$$

where $\omega_{\alpha \beta }$ is the weight value between the one-hot encoding layer and the embedding layer, and β is the index of the embedding layer. Now, we can see that the embedding of the mapping is merely the weights of this layer, which can be learned like the parameters of other layers of the neural network.

After representing all categorical variables using entity embedding, all embedding layers and continuous variable inputs are connected. The merged layer is considered a common input layer in the neural network. We obtain the embedding vectors from the trained entity embedding network and input them as part of the MLP features.

(2) Extraction of Speed Change Patterns on Continuous Sections Using LSTM

Continuous features in structured data, such as the speed of consecutive sections, typically exhibit characteristics of time series. The LSTM network is an excellent recurrent neural network that can proficiently capture and learn the time dependencies of continuous inputs. When a vehicle travels through several continuous sections, there may be certain patterns in its section travel speed. If several speed variables are directly used as model inputs, the neural network may fail to learn the patterns of speed changes. Therefore, we need to use LSTM networks to better understand the sequential characteristics of speed, such as acceleration, deceleration, or maintaining a constant speed. LSTM not only considers the speed of a single segment but also takes into account the speed changes in adjacent segments, providing the model with more contextual information, which is very valuable for analyzing vehicle behavior.

**Figure 8:**
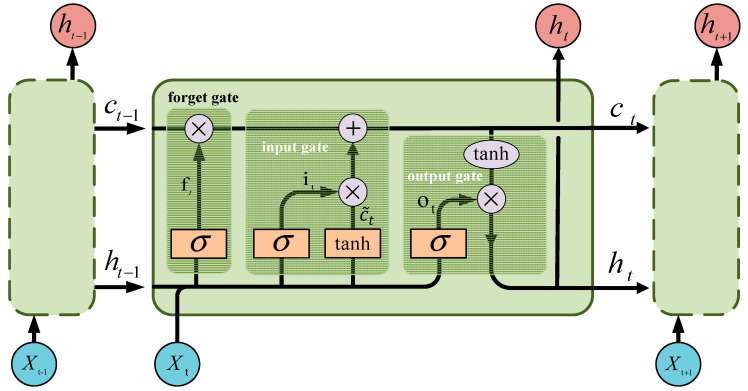
Principles diagram of LSTM network.

Each LSTM unit has a memory cell that is controlled by three gates: the input gate, forget gate, and output gate, as shown in Figure 8.

In Figure 8, $x_{t}$ represents the unit input at time t. $h_{t-1}$ and $h_{t}$ denote the hidden layer states at times t−1 and t, respectively. $c_{t-1}$ and $c_{t}$ represent the cell states at times t−1 and t. ${\tilde{c}}_{t}$ stands for the intermediate quantity during the calculation. σ denotes the sigmoid function, and tanh represents the tanh function. $i_{t}$ indicates the input gate. $f_{t}$ designates the forget gate. $o_{t}$ refers to the output gate. The process of LSTM extracting the changing patterns of speed variables is as follows:

Step 1: LSTM selectively forgets the characteristic information of cell state $c_{t-1}$ at moment t:

(18) $$f_{t}=\sigma \left(W_{xf}x_{t}+W_{hf}h_{t-1}+b_{f}\right)$$

(19) $$c_{t}^{\prime }=f_{t}⊙c_{t-1}$$

Step 2: LSTM selects important information from the input features to update the state unit $c_{t}^{\prime }$:

(20) $$\tilde{c_{t}}=tanh\left(W_{x\tilde{c}}x_{t}+W_{h\tilde{c}}h_{t-1}+b_{\tilde{c}}\right)$$

(21) $$i_{t}=\sigma \left(W_{xi}x_{t}+W_{hi}h_{t-1}+b_{i}\right)$$

(22) $$c_{t}=c_{t}^{\prime }+i_{t}⊙\tilde{c_{t}}$$

Step 3: Determining the output of LSTM:

(23) $$o_{t}=\sigma \left(W_{xo}x_{t}+W_{ho}h_{t-1}+b_{o}\right)$$

(24) $$h_{t}=o_{t}⊙tanh\left(c_{t}\right)$$

In the above formulas, ${W_{xi},\;W}_{xf},W_{xo}\in R^{m*n}$ represent the weight matrices of the current unit input $x_{t}$ for the input gate, forget gate, and output gate, respectively. ${W_{hi},W}_{hf},W_{ho}\in R^{m*m}$ represent the weight matrices of the previous hidden layer state $h_{t-1}$ for the input gate, forget gate, and output gate, respectively.$b_{i},b_{f},b_{o}\in R^{m}$ respectively represent the bias values for the input gate, forget gate, and output gate. We obtain the speed variation features from the trained LSTM network, and use them as inputs for the speed variation part of the MLP.

(3) Travel Time Estimation Using MLP

After processing the respective feature sets through the EENN and LSTM networks, we integrated them with other features to form a comprehensive feature vector. This can provide a more holistic information foundation for travel time estimation. We serve it as an input, through a simple three-layer MLP to estimate the travel time, further completing the time restoration of ETC missing transactions. Generally speaking, traditional methods might employ a singular model, treating all features in the same manner. But we chose the best processing method based on the properties of the features. This differentiated processing strategy accentuates the latent information within the data, further increasing the utilization rate of data.

## 4. Experiment

### 4.1. Experimental Conditions

#### 4.1.1. Data Source

**Figure 9:**
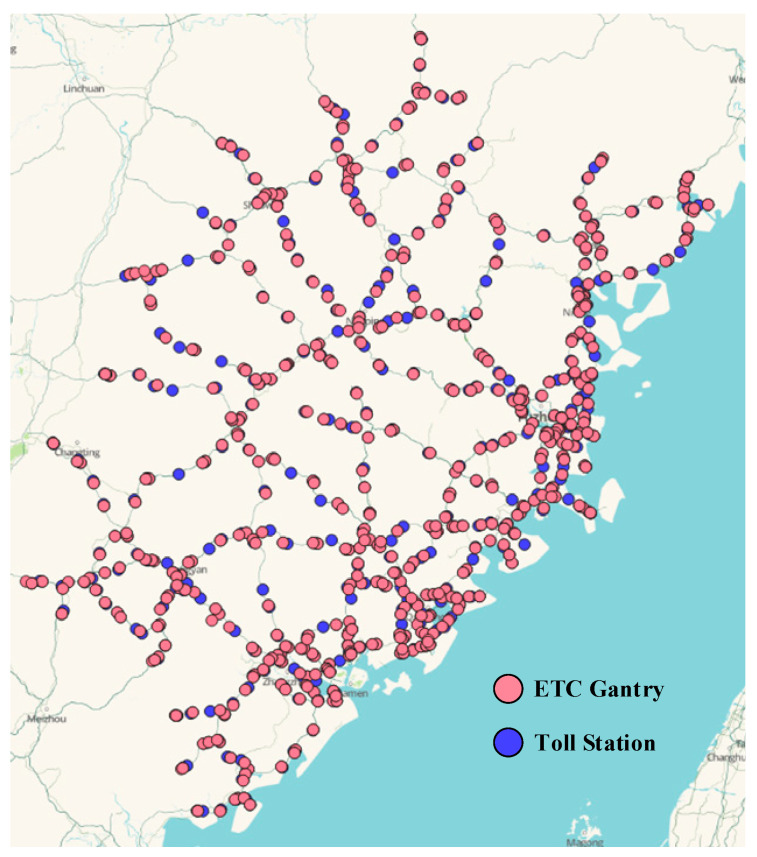
Map of the ETC gantry system setup in Fujian province.

This experiment selected data collected by the ETC gantry system throughout Fujian province on 1 May and 1 June 2021. The layout of the gantries is shown in Figure 9. After data preprocessing, we used a total of 3,986,845 pieces of trajectory data, with 80% as the training set and 20% as the test set.

#### 4.1.2. Equipment

The experiment was conducted on a computer equipped with an Intel Core i7-9900K (8 cores, 3.6 GHz), supplemented with 2 NVIDIA GeForce RTX 3060 graphics cards and 32GB DDR4 RAM. The primary programming language was Python 3.9, operating under the Windows 11 OS. The deep learning models were constructed using the PyTorch (version 2.0.1, optimized for CUDA 11.8) framework. The data processing was implemented in SQL on Clickhouse 21.11.4.14.

#### 4.1.3. Baseline Model

For the time restoration issue of ETC missing transaction data, the model proposed in this paper was compared with the following baseline methods commonly used for structured data regression tasks:

(1) Tabular Data Networks (TabNet): A deep learning model specifically designed for structured data. It incorporates attention mechanisms and some decision tree concepts to offer better interpretability and higher performance.
(2) Residual Networks (ResNet): Adds “residual connections” to reduce gradient vanishing and explosion problems during deep network training. Utilizing residual connections facilitates capturing long-term dependencies and complex patterns in the data.
(3) Transformer: Initially used for natural language processing tasks, it utilizes self-attention mechanisms to capture dependencies in sequential data, and can also be applied to structured data, especially when there are complex interdependencies in the data.
(4) Neural Decision Trees (NDT): A model that combines decision trees with neural networks to achieve the interpretability of decision trees and the performance of neural networks, very suitable for regression problems of structured data.
(5) Deep Feedforward Network (DFN): A fundamental deep neural network that can be used for regression problems of structured data, provided that feature engineering is handled properly.
(6) Random Forest (RF): An ensemble learning method composed of multiple decision trees that performs predictions through voting or averaging, exhibiting excellent performance in handling structured data, particularly when there are nonlinear relationships between features.
(7) K-Nearest Neighbor (KNN): An instance-based learning method, where predictions are based on the nearest neighbors in the feature space of the input sample, suitable for structured data but may degrade in performance with high-dimension data or large datasets.
(8) Decision Tree (DT): A tree-shaped model used for classification and regression, which is easy to interpret and highly applicable to structured data but tends to overfit.
(9) XGBoost: A high-performance gradient boosting tree model that excels in processing structured data, a preferred choice in many Kaggle competitions and industrial applications.
(10) LightGBM: A gradient boosting tree model that is lighter and more efficient, optimized for large-scale structured data, offering high computational efficiency.
(11) CatBoost: Another gradient boosting tree model, notable for its performance with structured data featuring categorical attributes.
(12) Linear Regression (LR): The simplest regression model, applying a linear equation to the data, suitable for issues where the relationships are approximately linear in structured data.
(13) Ridge Regression (RR): A regularized version of linear regression, used to handle collinearity and prevent overfitting. A good choice when structured data have multicollinearity.
(14) Lasso Regression: Another regularized linear regression that performs feature selection through the addition of an L1 regularization term, offering both regularization and feature selection functionalities, making it suitable for sparse feature selection in structured data.

#### 4.1.4. Evaluation Indicators

To evaluate the restoration performance of the models, the differences between the observed values $y_{i}$ and the estimated values ${\hat{y}}_{i}$ were assessed using the root mean square error (RMSE), the mean absolute error (MAE), and the coefficient of determination ($R^{2}$). The calculation formulas for the three evaluation metrics are as follows:

(25) $$R_{RMSE}\left(y,\hat{y}\right)=\sqrt{\frac{1}{T}\sum_{i=1}^{T}{\left(y_{i}-{\hat{y}}_{i}\right)}^{2}}$$

(26) $$M_{MAE}\left(y,\hat{y}\right)=\frac{1}{T}\sum_{i=1}^{T}\left|y_{i}-{\hat{y}}_{i}\right|$$

(27) $$R_{R^{2}}\left(y,\hat{y}\right)=1-\frac{\sum_{i}{\left(y_{i}-{\hat{y}}_{i}\right)}^{2}}{\sum_{i}{\left(y_{i}-\bar{y}\right)}^{2}}$$

#### 4.1.5. Model Parameters

The hyperparameters in the model were determined during the training process, which means that we selected the model with the best performance on the test set through MAE. Initially, hyperparameter ranges were manually set based on experience: initial learning rate {0.01,0.005,0.001,0.0005}, batch {16,32,64,128,256}, epoch {20,50,100,200}. For this model, the following settings were found to be the most effective: a learning rate set to 0.001, a batch size of 32, and epoch set to 100. These settings remained effective when using the baseline model. All deep learning models were implemented through the PyTorch framework and trained using the Adam optimizer. After multiple training sessions, the final model framework parameters were confirmed as shown in Table 3, which lists the model layers, node numbers, output sizes, and relevant hyperparameters.

### 4.2. Result Analysis

#### 4.2.1. Restoration Effect Comparison Experiment

**Figure 10:**
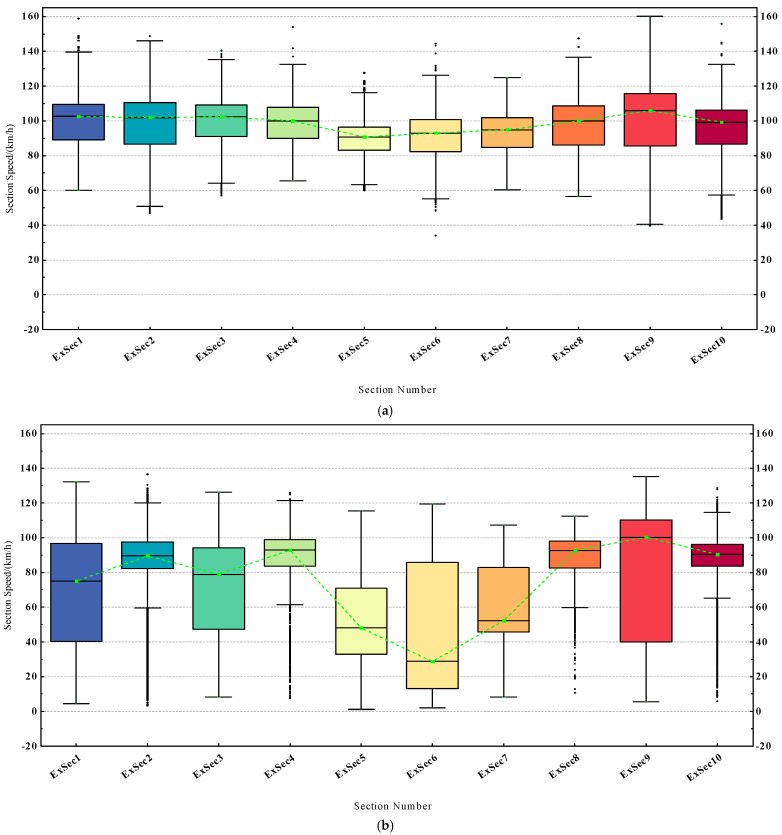
The vehicle section speed distribution box plot for 10 different sections in 24 h of a day, where (**a**) represents the non-holiday dataset, namely June 1st, and (**b**) represents the holiday dataset, namely May 1st.

Under normal circumstances, the traffic flow on expressways in various sections does not reached a saturated state. At the same time, under good road conditions, vehicles can generally travel smoothly. Since such driving conditions account for the majority of cases, our model needs to exhibit excellent performance when carrying out time restoration on datasets generated under these driving conditions. During non-holidays, the traffic flow on the expressways is less, and the datasets generated conform to this feature. We chose the ETC trajectory dataset from June 1st to represent the non-holiday dataset. Due to unforeseen circumstances such as traffic accidents and road maintenance, expressways often experience traffic congestion, especially during holidays when the demand for travel increases, exacerbating the congestion phenomenon. Severe traffic congestion can affect a long stretch of road, causing substantial fluctuations in section speed over two or even more consecutive sections. We chose the trajectory dataset of May 1st to represent the holiday dataset. As shown in Figure 10, the box width difference indicates that the range of vehicle speeds in each section during the holidays is large, leading to a higher degree of data dispersion. From the position of the median, it can be seen that, compared to the similar speed distribution range between sections during non-holidays, the speed distribution differences between sections during holidays were larger. There are also more outliers in some sections, which still implies a higher possibility of traffic congestion during the holidays.

The restoration effects of the various models in the non-holidays dataset are shown in Table 4, where the bold part represents the optimal value. It should be noted that all models used the same dataset and feature selection and were transformed into different input forms as restored by the models.

It can be seen that the $R^{2}$ values of all the models are very close to 1, indicating that these models performed very well in terms of data fitting. However, the task of time restoration of missing transactions data in expressway ETC requires not only that the model can perfectly interpret the data but also maintain low restoration errors, along with small fluctuations in restoration errors when facing outliers. The linear models represented by LR were far less effective in restoration compared to other baseline models, with the best performance metrics being MAE, RMSE, and $R^{2}$ values of 31.319, 49.103, and 0.972, respectively. Compared to the linear models, the traditional machine learning and deep learning models performed better, owing to their ability to better capture non-linear relationships in the data. Hence, current data mining research is gradually inclined towards using these models. Overall, the models based on gradient boosting trees stand out, among which Xgboost had the best performance with MAE, RMSE, and $R^{2}$ values of 15.312, 26.657, and 0.991, respectively. The model we proposed improved the restoration effect compared to Xgboost, with an increase in MAE and RMSE by 19.06% and 10.66%, respectively, making all the evaluation metrics the best.

**Figure 11:**
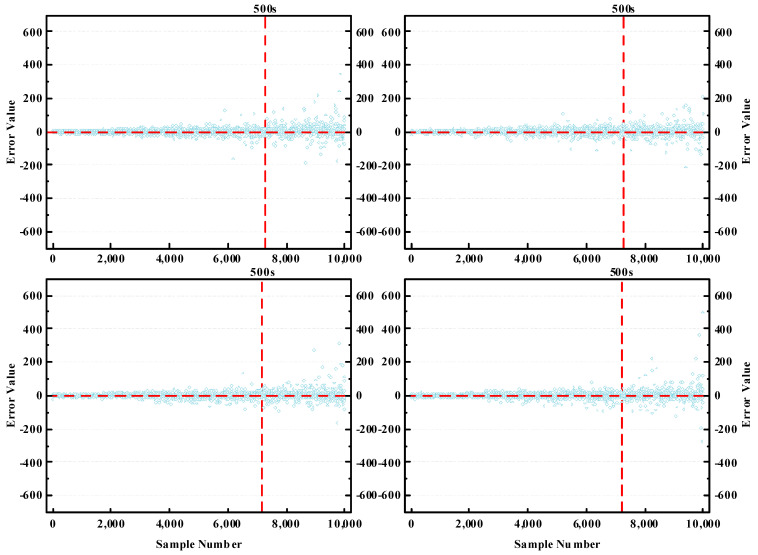
Error degree between estimated value and actual value.

To more clearly demonstrate the restoration effect of the model, we constructed scatter plots showing the error degree between the estimated and actual values. Given the vast size of the test dataset, we carried out four random samplings of the test data, taking 10,000 data points each time, as shown in Figure 11.

As can be seen, the time restoration effect is generally better for sections with a travel time of less than 500s. For sections with longer travel times, the fluctuations in time restoration errors are more pronounced. When there is no congestion, the long travel time for vehicles in the section implies a longer section distance. Compared with short distance sections, the driving state of long-distance sections changes more complexly, making the driving patterns harder to capture. Even so, our model still demonstrated strong performance in restoration accuracy. Furthermore, since the proportion of sections with longer travel times was lower in the dataset, the overall restoration effect of the model was very good.

The holiday dataset had a larger volume of data, and its complexity was also higher. Even though we pay more attention to the time restoration during periods when the traffic flow is not in a saturated state, we still need to address the time restoration issue in the holiday dataset. Therefore, we compared the restoration performance of all models on this dataset, with the effects illustrated in Table 5.

It can be seen that the data fitting degree of the linear model was very low, with the optimal R^2^ value being only 0.461, and the best MAE and RMSE values being 150.380 and 464.810, respectively, indicating the high difficulty in time restoration for the holiday dataset. Generally speaking, the deep learning models had superior restoration effects compared to the tree-based machine learning models. This is primarily due to the deep learning model’s ability to capture complex data patterns more effectively, thereby displaying a more pronounced advantage in terms of time restoration. Our proposed model not only rivalled the Transformer model in MAE value, but also had the highest $R^{2}$ value and the lowest RMSE value, demonstrating the best overall restoration performance on the holiday dataset. This further reflects the stability and universal applicability of the model we proposed in dealing with time restoration tasks.

#### 4.2.2. Ablation Experiment

To analyze the role of various components of the model, further verify the efficacy of algorithm improvements, and enhance the model’s interpretability, we conducted ablation experiments. We successively removed the EENN modules and LSTM modules, and replaced the EENN’s handling of discrete variables with one-hot encoding processing. The effects of the ablation experiments are shown in Table 6.

It can be seen that utilizing the EENN to handle discrete variables enhanced the performance of the model, with the MAE and RMSE values improving by 7.60% and 4.68%, respectively, whereas using one-hot encoding to handle discrete variables conversely increased the errors in time restoration. This might be because one-hot encoding, while increasing the dimensionality of the dataset, does not offer much useful information about the discrete variables, easily leading to a very sparse feature matrix that is not conducive to model training and generalization. By using LSTM to extract the vehicle speed variation features over several continuous sections, we likewise improved the effect of time restoration, increasing the MAE and RMSE values by 9% and 7.29%, respectively. From the foregoing analysis, it can be inferred that for regression problems in structured data, it is essential to use appropriate methods or models to deal with different types of feature inputs to enhance the performance of the model and better address practical issues.

#### 4.2.3. Features Contribution Analysis

In this study, our model holistically considered four groups (comprising 11 individual features) for time restoration. The selection of these features was based on their anticipated significance and potential time restoration capacity, as well as preliminary data analysis. We employed these features to grasp the multifaceted influences affecting the estimations of vehicles’ section travel times. To evaluate the contribution of these features to the model’s estimation efficacy, we sequentially removed each group of features and monitored the impact on the model’s performance. Table 7 presents the results of the experiment.

From Table 6, we can observe that in the two distinct datasets, both driving behavior features and section geographical features made a notable contribution to the model. During non-holiday periods, the traffic flow in most sections did not reach saturation. Under such circumstances, the estimation of section travel times of vehicles relies more on the differences in individual driving behaviors. Additionally, the geographical features of the sections played a supplementary role. During holidays, the substantial traffic flow on expressways means that individual vehicle driving behavior might exert a larger influence on the overall traffic stream, making this feature still very important. Meanwhile, when traffic overflow leads to congestion, the geographical attributes of the sections become particularly crucial. For instance, amidst traffic congestion, more vehicles may opt to enter service areas to wait, unquestionably augmenting the section travel times for vehicles. Compared to the above two features, the contributions from traffic situation features and the vehicle classes feature are relatively low. The contribution of traffic condition features exhibits noticeable differences between the two datasets. This can be attributed to the unsaturated traffic flow in most sections during non-holiday periods, rendering it unable to affect the vehicles’ travel times significantly. However, during holidays, a higher number of sections experienced oversaturated traffic flow, creating more congestion scenarios, hence the traffic condition features reflect the transit status of vehicles in the sections to a certain extent. Although the vehicle classes feature holds a lower stake in feature contribution, being readily accessible information, it still has a positive effect on the model’s estimation accuracy to some degree.

## 5. Conclusions

Due to malfunctions of ETC equipment or abnormalities in signal transmission, missing transactions occur in ETC vehicle trajectory data. ETC data are not only vast in volume, but also complex in its composition. The existing methods cannot effectively address the ETC data restoration issue. So we proposed a method for restoring ETC data. Specifically, we first transformed the time restoration issue into a vehicle section travel time estimation problem and extracted four kinds of features for the estimation of travel time, which are section geographical features, the vehicle classes feature, driving behavior features, and traffic situation features. Next, we employed an EENN to automatically learn the representations of categorical features in multi-dimensional spaces, subsequently obtaining embedded vectors from the trained EENN, which were used as an input for the categorical features of the model. Following that, we used an LSTM to extract the changing patterns of vehicle speeds over several consecutive sections and inputted it as speed variation features. Lastly, combining the processed features with other features that do not require processing, we employed a MLP to complete the estimation of the vehicle section travel time, achieving the goal of time restoration. To verify the general applicability, stability, and accuracy of the above method, we selected the real ETC transaction datasets collected by the provincial ETC gantry system in Fujian on 1 May and 1 June 2021, as the experimental datasets. Comparative experimental results show that the method we proposed outperformed the selected baseline models on both datasets. Compared to the best baseline model, the MAE and RMSE values of the non-holiday dataset increased by 19.06% and 10.66%, respectively. This method is also fit for more complex ETC datasets, and it exhibited the best overall recovery performance on the holiday dataset. Its R2 reached 0.91, and the MAE and RMSE values were 56.81 and 189.778, respectively. The ablation test confirmed that each module of our method plays a significant positive role in enhancing data restoration accuracy. The EENN increased the MAE and RMSE values by 7.60% and 4.68%, while LSTM improved the MAE and RMSE values by 9% and 7.29%. Our method takes into account the multi-dimensional features that affect vehicle travel time. We not only used manual feature extraction to provide the model with meaningful and strongly correlated primary inputs, but also employed deep learning models to learn deeper representations from these features, capturing more intricate non-linear relationships and patterns. This method, which combines manual features with automatic feature learning, enhances the interpretability of deep learning models and increases data utilization. In addition, it can also reduce the model’s reliance on noise or irrelevant features, resulting in a more robust model post-training. The experiment demonstrated that this method can effectively restore ETC missing transaction data, improve the quality of expressway ETC transaction data, and promote the intelligent management and operation of expressways. It is worth noting that while the method is designed for restoring ETC data, its core ideas and strategies can be applied to other large-scale structured datasets similar to ETC data. However, in real life, road factors of different sections such as slope and curvature affect the driving speed of vehicles in sections, further affecting the missing transaction time restoration. Therefore, in future work, we will consider more features and influencing factors to further improve restoration accuracy. Simultaneously, we will contemplate the issue of online restoration of ETC missing transaction data. Overall, this study has achieved satisfactory results on ETC data restoration, and we believe that the proposed method holds potential value in a broader range of application scenarios, providing a new direction for future research.

## Figures and Tables

**Table 1:** Summary of related work.

Type	Method	Example	Advantages	Disadvantage
Statistical-based Restoration Methods	Historical Mean Interpolation	Local average [8], global average [9]	Simple and fast, easy to understand	Not suitable for nonlinear relationships and classification data, poor accuracy
	PCA	FPCA [12], PPCA [13], KPPCA [14]	Can reduce data dimensions, does not need labels	Not suitable for nonlinear relationships, poor interpretability, sensitivity to outliers
Relational-based Restoration Methods	Low-rank Matrix and Tensor Completion [15,16,17,18,19,20,21]	\	Fully utilizes data structure, fewer model parameters	High algorithm complexity, excessive reliance on single parameter selection for restoration
	Functional Dependencies and Conditional Constraints [22,23,24,25,26,27,28]	\	Simple and intuitive, does not need additional data and models	There are not always clear constraints, algorithm complexity is high, strict linear and equality relationships between data attributes
Learning-based Restoration Methods	Machine Learning	CA [29,30,31,32,33], XGBoost [34], RF [35]	The algorithm application is relatively mature and has strong interpretability	Requires a large amount of annotated data, poor adaptability to large datasets
	Deep Learning	GRNN [37], SA [38], LSTM [39], IGAN [40], GCN [41]	Suitable for processing complex patterns, strong adaptability to large datasets	Poor interpretability, high data volume requirements, high performance requirements

**Table 2:** Selected fields for ETC transaction data.

Number	Field Name	Field Attributes	Example
1	tradetime	transaction time	2021-05-01 00:00:00
2	flagid	gantry number	34**17
3	obusn	vehicle ID	3501********8316
4	vehclass	vehicle class	1
5	entime	entry time	2021-05-01 01:00:00
6	enstation	entrance station	1**1
7	extime	outbound time	2021-05-01 01:00:00
8	exstation	exit station	1**1
9	distance	section distance	1234
10	service	service area name	** service area
11	tunnel	number of tunnels	1
12	tunnel_distance	tunnel distance	1234

**Table 3:** Model parameter.

Model Components	Model Hyperparameter	Parameter Value
EENN	Hidden layer size N×D	Vehicle Class: 3×2 Time Zone: 96×48 Number of Tunnels: 14×7 RSA: 2×1
	Number of layers	4
LSTM	Hidden layer size × Number of layers	3×1
MLP	Hidden layer size × Number of layers	128×1 64×1 32×1 1×1
Overall model	Batch size Learning rate Epoch Optimization methods	32 0.001 100 Adam

**Table 4:** Time restoration effects of different models on non-holiday dataset.

Model Name	MAE	RMSE	$R^{2}$
TabNet	16.313	30.569	0.989
RstNet	18.259	34.932	0.986
Transformer	16.884	31.741	0.988
NDT	21.702	38.857	0.982
DFN	17.441	33.395	0.987
RF	17.666	35.444	0.985
DT	24.552	49.261	0.972
KNN	19.523	38.624	0.982
XGBoost	15.312	26.657	0.991
LightGBM	17.570	31.688	0.988
Catboost	17.618	31.206	0.988
LR	31.319	49.103	0.972
RR	31.369	49.255	0.971
Lasso Regression	31.361	49.268	0.972
**Our Model**	**12.394**	**23.815**	**0.993**

**Table 5:** Time restoration effects of different models on holiday dataset.

Model Name	MAE	RMSE	$R^{2}$
TabNet	61.426	200.748	0.899
RstNet	68.052	216.758	0.882
Transformer	53.138	222.1	0.876
NDT	105.136	343.818	0.705
DFN	64.4394	206.152	0.893
RF	107.462	448.371	0.498
DT	127.509	617.78	0.47
KNN	105.135	457.158	0.478
XGBoost	109.408	433.242	0.532
LightGBM	111.952	433.526	0.531
Catboost	114.016	433.532	0.529
LR	150.380	470.223	0.455
RR	150.731	464.810	0.461
Lasso Regression	150.399	464.829	0.460
**Our Model**	56.81	**189.778**	**0.91**

**Table 6:** Ablation experiment.

Module Name	MAE	RMSE	$R^{2}$
MLP	14.739	26.949	0.991
MLP + EENN	13.620	25.689	0.992
MLP + One-Hot	15.890	28.955	0.990
MLP + EENN + LSTM	12.394	23.815	0.993

**Table 7:** Evaluation of feature contribution experimental results.

Feature Set	Non-Holiday Dataset		Holiday Dataset	
	MAE	RMSE	MAE	RMSE
$\left(\varepsilon ,\eta ,\theta \right)$	20.256	41.145	93.769	285.899
$\left(\gamma ,\eta ,\theta \right)$	14.809	26.301	57.262	**185.287**
$\left(\gamma ,\varepsilon ,\theta \right)$	28.139	48.025	120.698	443.987
$\left(\gamma ,\varepsilon ,\eta \right)$	13.635	25.6967	66.733	189.343
$\left(\gamma ,\varepsilon ,\eta ,\theta \right)$	**12.394**	**23.815**	**56.81**	189.778

## Data Availability

The ETC transaction data utilized in this study were obtained from Fujian Expressway Information Technology Co., Ltd. (Fuzhou, China). Restrictions apply to the availability of these data, which were used under license for this study and are not publicly available. Data are available from the authors with the permission of Fujian Expressway Information Technology Co., Ltd. All data processing and analyses were conducted in compliance with relevant data protection and privacy laws. No individual or personal data were used in this study.
